# Splenomegaly in de novo acute myeloid leukemia is associated with *ASXL1* mutations together with a distinct clinical and gene expression profile

**DOI:** 10.1186/s40364-025-00833-8

**Published:** 2025-10-22

**Authors:** Francesco Tarantini, Nicoletta Coccaro, Cosimo Cumbo, Immacolata Redavid, Luisa Anelli, Antonella Zagaria, Elisa Parciante, Maria Rosa Conserva, Giuseppina Tota, Crescenzio Francesco Minervini, Angela Minervini, Mariano Francesco Caratozzolo, Flaviana Marzano, Claudia Telegrafo, Bachir Balech, Anna Mestice, Vito Pier Gagliardi, Mario Delia, Paola Carluccio, Maria Giovanna Macchia, Apollonia Tullo, Giorgina Specchia, Pellegrino Musto, Francesco Albano

**Affiliations:** 1 Department of Precision and Regenerative Medicine, Ionian Area - DiMePRe-J - Hematology and Stem Cell Transplantation Unit, University of Bari “Aldo Moro”, P.Zza G. Cesare, 11, 70124 Bari, Italy; 2 Institute of Biomembranes, Bioenergetics and Molecular Biotechnologies, IBIOM - National Research Council - CNR, Bari, Italy; 3 School of Medicine, University of Bari “Aldo Moro”, Bari, Italy

**Keywords:** Acute myeloid leukemia, Splenomegaly, *ASXL1*, *PCDHB2*, *LURAP1L/LURAP1L-AS1*

## Abstract

**Background:**

Splenomegaly is an event occurring in a variable range between 10–40% of de novo acute myeloid leukemia (AML), recently linked to poorer prognosis. Studies in murine models have shown that loss of the additional sex combs-like 1 *(ASXL1)* gene function leads to a significantly enlarged spleen volume, due to an increased infiltration of myeloid cells into the spleen.

**Methods:**

In 58 de novo AML patients presenting with splenomegaly at diagnosis, we evaluated the occurrence of *ASXL1* somatic mutations, deepened the molecular profile and conducted high-throughput RNA sequencing, with the aim of unveiling possible peculiar aspects of this rare clinical scenario.

**Results:**

*ASXL1* mutations (*ASXL1*mut) were detected in 23/58 (40%) patients, being the most frequently mutated gene, followed by *TET2* and *NRAS*. *ASXL1*mut cases were significantly older than *ASXL1*wt (71 vs 64 years old, p = 0.003), showed a significantly higher white blood cells count (31,970/uL vs 17,810/uL, p = 0.044) and a higher platelet count (177,700/uL vs 67,700/uL, p = 0.0006). In contrast, the median bone marrow blasts percentage was lower in the *ASXL1*mut subset compared to *ASXL1*wt (36.4% vs 72,1%, p = 0.002). Comparing the gene expression profile of the *ASXL1*mut and *ASXL1*wt groups, we found the upregulation of *PCDHB2* and *LURAP1L/LURAP1L-AS1* (all involved in mechanisms of cellular interaction and migration) genes in the former group, unveiling a role in the splenic infiltration of *ASXL1*mut leukemic cells.

**Conclusions:**

Overall, our data paves the way for further studies of an AML subgroup with a distinctive phenotype, whose prompt identification could improve patient management and therapeutic decision making.

**Supplementary Information:**

The online version contains supplementary material available at 10.1186/s40364-025-00833-8.

## To the Editor

Splenomegaly is a pathological enlargement of the spleen, whose prevalence in the context of acute myeloid leukemia (AML) varies, with studies reporting its occurrence in 10–40% of cases [1, 2]. A thorough characterization of biological and clinical features of de novo AML patients presenting with an enlarged spleen volume at diagnosis is lacking. Studies in murine models have shown that loss of *ASXL1* function leads to significant splenomegaly; histological analyses of spleens from *ASXL1*-mutant mice demonstrate a disorganized architecture and increased presence of myeloid cells, highlighting the *ASXL1* role in maintaining normal spleen function and its contribution to pathological spleen enlargement in myeloid malignancies [3].

In 58 de novo AML patients with splenomegaly at diagnosis (Supplementary Table 1), we evaluated the occurrence of *ASXL1* somatic mutations, with the aim of unveiling possible biological and phenotypical peculiar aspects of this uncommon clinical scenario (see methodological approaches in Supplementary File 1).

**Fig. 1:**
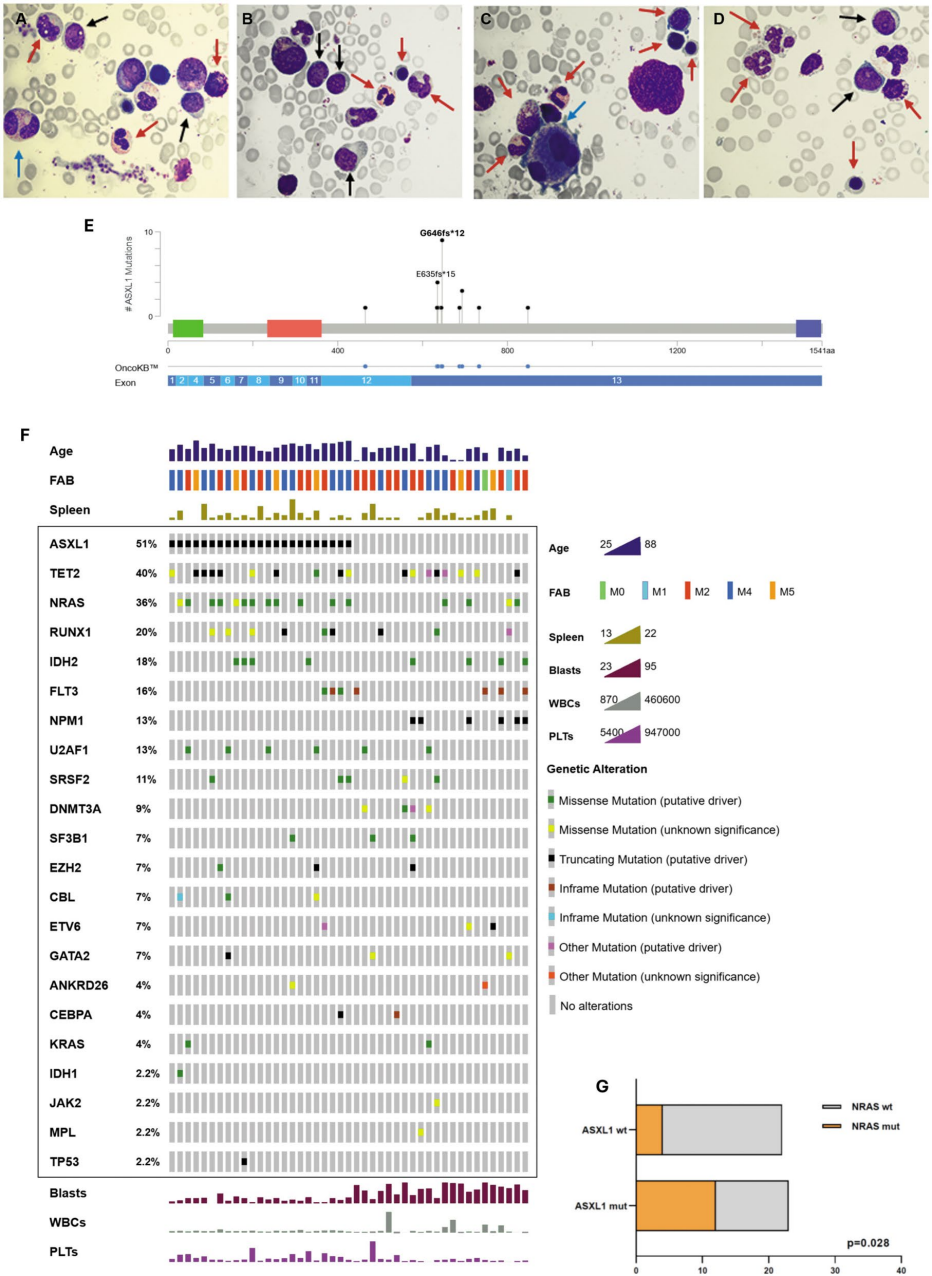
Morphological assessment and mutational analysis. Bone marrow smears from an *ASXL1*mut patient showing the coexistence of both normal mature and pathologic (dysplastic, blast) cells. Red arrows indicate normal myeloid and lymphoid cells. Blue arrows indicate dysplastic cells. Black arrows indicate blasts. **A** from left to right, red arrows indicate a monocyte, a myelocyte, a neutrophilic granulocyte; blue arrow indicates a dysplastic myelocyte; black arrows indicate a monoblast and a myeloblast. **B** red arrows indicate a neutrophilic granulocyte, a late erythroblast, a monocyte; black arrows indicate three myeloblasts. **C** red arrows indicate a band cell, a promyelocyte, another band cell, a basophilic erythroblast, a lymphocyte and a late erythroblast; the blue arrow indicates a dysplastic megakaryocyte. **D** red arrows indicate two monocytes, a late erythroblast and a myelocyte; black arrows indicate two myeloblasts. **E** Map of *ASXL1* variants identified on a linear schematization of the protein (lollipop plots) (hg19, RefSeq: NM_015338). **F** Oncoprinter visualization of all variants identified. For all cases (columns), main clinical parameters are reported. The percentage value reported for each gene indicates its variants occurrence in the cohort analyzed. **G** *NRAS* mutational profile. FAB: French-American-British classification, WBCs: white blood cells, PLTs: platelets

Splenomegaly was detected in 58/560 (10,4%) patients diagnosed with de novo AML at our institution from 2005 to 2022. A certain degree of both myeloid and erythroid maturation was observed alongside the presence of blasts (Fig. 1A-D). Although this finding may occasionally occur in AML in general, in our series it represents a recurrent cytomorphological signature.

*ASXL1* mutations were detected in 23/58 (40%) patients, with a variant allele frequency (VAF) ranging from 10 to 80%; all of them had a truncating effect: 20 (87%) were frameshift insertions/deletions and 3 (13%) nonsense variants affecting exons 12 and 13 (as widely described in AML) [4]. The most frequent variant was p. G646fs*12 (8/23, 35%) followed by p. E635fs*15 (4/23, 17%) (Fig. 1E). Most mutations (21 out of 23) produced truncated ASXL1 proteins that lack the C-terminal PHD domain but can exert a neomorphic activity by disrupting Polycomb repression and enhancing BAP1 deubiquitinase activity [4].

Upon molecular characterization of our AML cases with splenomegaly, a total of 146 variants were identified across 22 of the 26 genes commonly mutated in myeloid neoplasms [5, 6] (Fig. 1F). *ASXL1* was the most frequently mutated gene (23/45, 51%), followed by *TET2* (18/45, 40%) and *NRAS* (16/45, 36%). Interestingly, the occurrence of *NPM1* and *TP53* mutated cases (representing two distinct AML subclasses) is largely under-represented in our cohort (Fig. 1F).

Moreover, a statistically significant association was observed between *ASXL1* and *NRAS* mutations. Indeed, 12/23 (52%) *ASXL1*mut cases had concurrent *NRAS* mutations, compared to 4/22 (18%) *ASXL1*wt cases (p = 0.028 – Fig. 1G). The cooperative leukemogenic interaction between *ASXL1* mutations and activating *NRAS* mutations was reported in myeloid malignancies, and this co-occurrence was associated with poorer outcomes [7]. In mice, *ASXL1* loss with oncogenic *NRAS* G12D mutation led to hyperactivation of RAS-MAPK signalling via *FLT3* upregulation**,** induction of *AP1* oncogenic programs, immune microenvironment suppression, increased spleen weights and promoted progression from chronic myelomonocytic leukemia to AML [8].

**Fig. 2:**
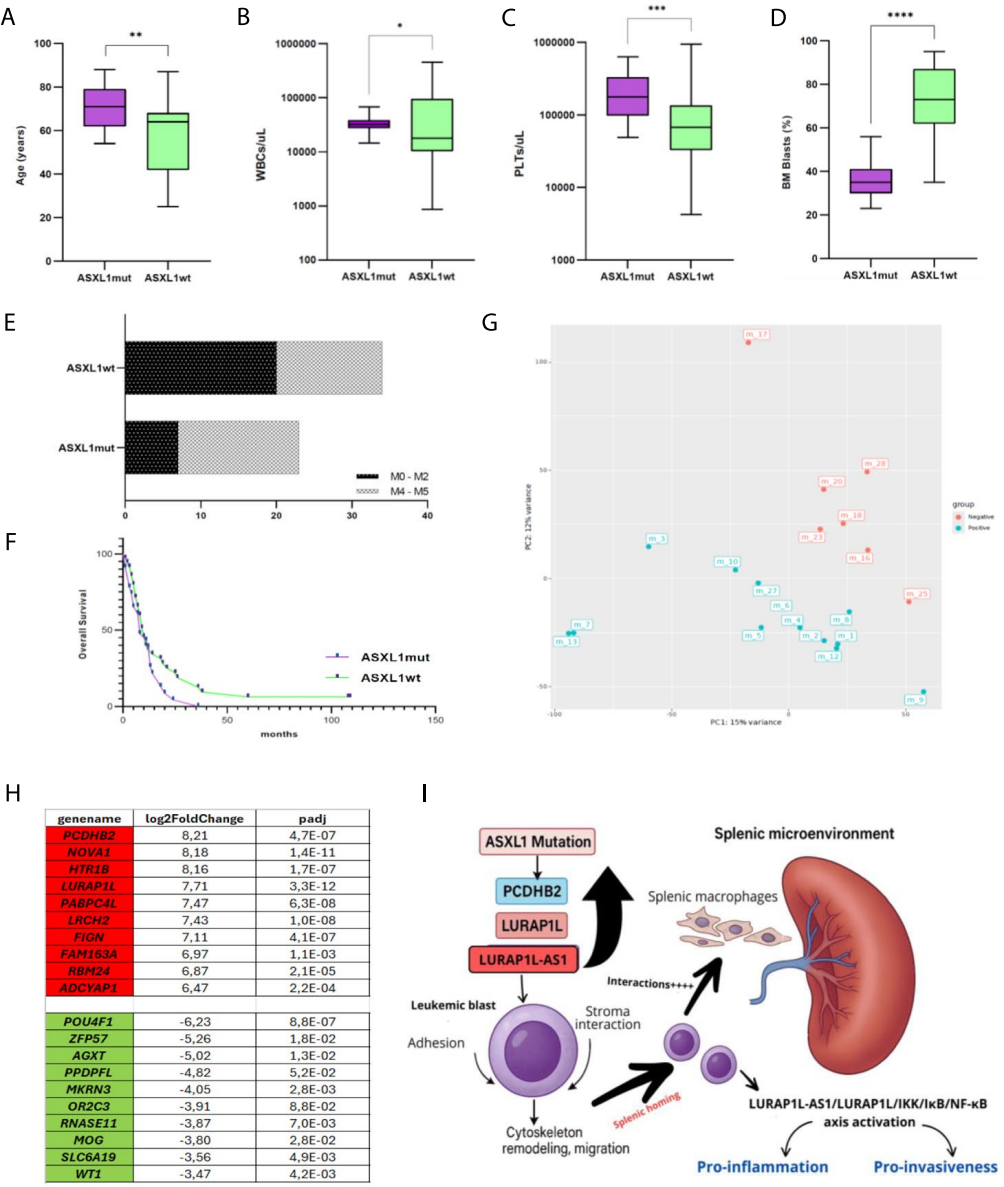
*ASXL1*mut vs *ASXL1*wt comparisons. Comparisons between *ASXL1*mut and *ASXL1*wt subgroups. Main clinical and biological parameters are reported: (**A**) Age, (**B**) WBCs, (**C**) PLTs, (**D**) BM blasts percentage, (**E**) FAB subtypes, (**F**) Overall survival. **A-D** Boxplots representing the distribution. The boxes extend from the 25th to 75th percentiles. The line in the box represents the median value. The whiskers range from the smallest value to the largest. **G** Principal Component Analysis (PCA) showing the separation between the two groups of samples (*ASXL1*wt and *ASXL1*mut) and highlighting the homogeneity of samples within each group. **H** Top ten differentially expressed protein-coding genes [upregulated (in red) and downregulated (in green)]. **I** Illustration of a possible mechanism connecting *ASXL1* mutations and splenomegaly in AML, based on RNA seq results and recent literature data. In leukemic cells, the *ASXL1* loss of function could induce the upregulation of *PCDHB2* and of the couple *LURAP1L/LURAP1L-AS1* (all involved in mechanisms of cellular interaction and migration). This could play a role in the splenic infiltration of *ASXL1*mut leukemic cells

Our cohort was consequently divided into two subsets: those bearing *ASXL1*mut (23/58, 40%) and those *ASXL1*wt (35/58, 60%). *ASXL1*mut cases were significantly older than *ASXL1*wt (71 vs 64 years, p = 0.003) (Fig. 2A), had higher total white blood cells count (31,970/uL vs 17,810/uL, p = 0.044) (Fig. 2B) and platelet count (177,700/uL vs 67,700/uL, p = 0.0006) (Fig. 2C). The median bone marrow blasts percentage was lower in the *ASXL1*mut subset compared to the *ASXL1*wt (36.4% vs 72.1%, p = 0.002) (Fig. 2D). A trend towards a myelomonocytic and monocytic differentiation was observed in the *ASXL1*mut patients [M4-5 = 16/23 (70%) in the *ASXL1*mut subset vs 14/34 (41%) in the *ASXL1*wt subset, p = 0.057 – Fig. 2E]. Survival analysis showed no differences between the two groups (p = 0.11, Fig. 2F).

Gene expression profiles from 13/23 *ASXL1*mut cases (with splenomegaly) were compared with 7 *ASXL1*wt patients (4 with and 3 without splenomegaly). According to the Principal Component Analysis (PCA) results, carried out on read counts relative to the expressed genes, a clear separation was observed between the two groups (Fig. 2G). A detailed description of RNAseq data analysis is reported in Supplementary File 1. *PCDHB2* gene resulted the TOP UPregulated between differentially expressed genes (DEGs) (Log2FC = 8.211 and p-adjusted value 4.74e-07) and both the couple *LURAP1L* and *LURAP1L-AS1* resulted upregulated (7.71 and 7.97 times) in *ASXL1*mut patients (Fig. 2H).

Interestingly, *PCDHB2*, *LURAP1L*, and *LURAP1L-AS1* are all involved (via distinct mechanisms) in processes of adhesion/migration and cell-stroma/immunity interactions [9–11]. Their aberrant overexpression in *ASXL1*mut blasts might therefore contribute to a biological phenotype favoring their splenic infiltration (Supplementary Fig. 1). To date, a role in promoting extramedullary infiltration in AML has been demonstrated only for miR-29c&b2 [12] but the lack of data on microRNA expression in our samples does not allow us to verify its possible role in our cohort. Acknowledging the limitations of this study, we recognize that no functional analyses were performed to validate the mechanistic impact of *ASXL1* mutations or the involvement of candidate effector genes. However, our primary objective was to identify and characterize a distinct AML subgroup with splenomegaly and a unique clinical-molecular profile. Future studies will be required to functionally dissect the pathogenic mechanisms suggested by our findings and to better define the biological and clinical relevance of *NRAS* mutations in the context of *ASXL1*-mutated AML patients with splenomegaly.

In conclusion, we describe a subset of AML patients characterized by splenomegaly, with reproducible clinical features and a genomic profile enriched for *ASXL1* mutations. The possible pathogenic involvement of novel players paves the way for deeper investigation. Future studies will be needed to clarify or refine this small but interesting piece of the AML puzzle.

## Supplementary Information

Supplementary Material 1. File 1.

Supplementary Material 2: Figure 1.

Supplementary Material 3: Figure 2.

Supplementary Material 4: Table 1.

Supplementary Material 5: Table 2.

Supplementary Material 6: Table 3.

Supplementary Material 7: Table 4.

Supplementary Material 8: Table 5.

Supplementary Material 9: Table 6.

Supplementary Material 10: Table 7.

## Data Availability

Data is provided within the manuscript or supplementary information files.

## Abbreviations

AML: Acute myeloid leukemia
VAF: Variant allele frequency
PCA: Principal Component Analysis
DEGs: Differentially expressed genes
