# Supplementary material for: Splenomegaly in de novo acute myeloid leukemia is associated with ASXL1 mutations together with a distinct clinical and gene expression profile
Source: Biomark Res. 2025 Oct 22;13:131. doi: 10.1186/s40364-025-00833-8 (PMC12542016; doi:10.1186/s40364-025-00833-8)
Supplement: Supplementary file 1 — Supplementary Material 1. File 1. [file 40364_2025_833_MOESM1_ESM.pdf]

A

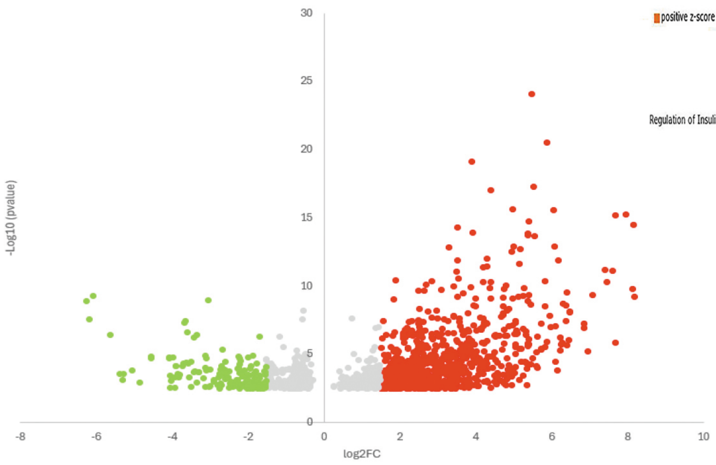

C

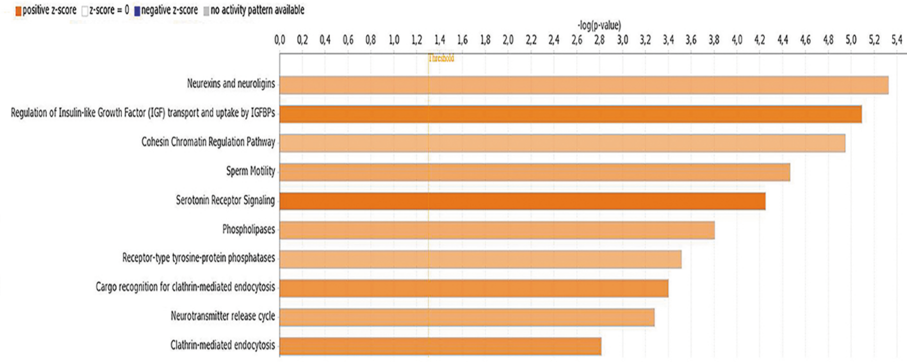

B

Category	Term	Count	%	PValue
GOTERM_BP_DIRECT	GO:0007155~cell adhesion	42	9,3	2,60E-13
GOTERM_BP_DIRECT	GO:0007399~nervous system development	37	8,2	5,41E-13
GOTERM_BP_DIRECT	GO:0007156~homophilic cell adhesion via plasma membrane adhesion molecules	23	5,1	6,51E-11
GOTERM_BP_DIRECT	GO:0016339~calcium-dependent cell-cell adhesion via plasma membrane cell adhesion molecule	10	2,2	1,39E-06
GOTERM_BP_DIRECT	GO:0007268~chemical synaptic transmission	17	3,8	3,30E-05
GOTERM_BP_DIRECT	GO:0007416~synapse assembly	9	2,0	7,62E-05
GOTERM_BP_DIRECT	GO:0007275~multicellular organism development	9	2,0	2,92E-04
GOTERM_BP_DIRECT	GO:0001525~angiogenesis	16	3,6	3,63E-04
GOTERM_BP_DIRECT	GO:0010628~positive regulation of gene expression	24	5,3	4,90E-04
GOTERM_BP_DIRECT	GO:0014059~regulation of dopamine secretion	6	1,3	4,95E-04
GOTERM_CC_DIRECT	GO:0005886~plasma membrane	185	41,1	4,23E-13
GOTERM_CC_DIRECT	GO:0009986~cell surface	32	7,1	1,84E-05
GOTERM_CC_DIRECT	GO:0031012~extracellular matrix	15	3,3	4,92E-04
GOTERM_CC_DIRECT	GO:0030669~clathrin-coated endocytic vesicle membrane	8	1,8	6,47E-04
GOTERM_CC_DIRECT	GO:0005911~cell-cell junction	13	2,9	6,99E-04
GOTERM_CC_DIRECT	GO:0062023~collagen-containing extracellular matrix	20	4,4	8,90E-04
GOTERM_CC_DIRECT	GO:0098978~glutamatergic synapse	20	4,4	0,001697293
GOTERM_CC_DIRECT	GO:0045202~synapse	22	4,9	0,001996106
GOTERM_CC_DIRECT	GO:0030425~dendrite	20	4,4	0,003471207
GOTERM_CC_DIRECT	GO:0002116~semaphorin receptor complex	4	0,9	0,00478402
GOTERM_MF_DIRECT	GO:0005509~calcium ion binding	51	11,3	6,59E-13
GOTERM_MF_DIRECT	GO:0005201~extracellular matrix structural constituent	10	2,2	8,98E-04
GOTERM_MF_DIRECT	GO:0017154~semaphorin receptor activity	4	0,9	0,003603154
GOTERM_MF_DIRECT	GO:0016597~amino acid binding	4	0,9	0,003603154
GOTERM_MF_DIRECT	GO:0005102~signaling receptor binding	17	3,8	0,005931186
GOTERM_MF_DIRECT	GO:0004064~arylesterase activity	3	0,7	0,006409076
GOTERM_MF_DIRECT	GO:0008013~beta-catenin binding	8	1,8	0,009192752
GOTERM_MF_DIRECT	GO:0050839~cell adhesion molecule binding	6	1,3	0,016687603
GOTERM_MF_DIRECT	GO:0048018~receptor ligand activity	6	1,3	0,017647967
GOTERM_MF_DIRECT	GO:0003779~actin binding	13	2,9	0,020555701

D

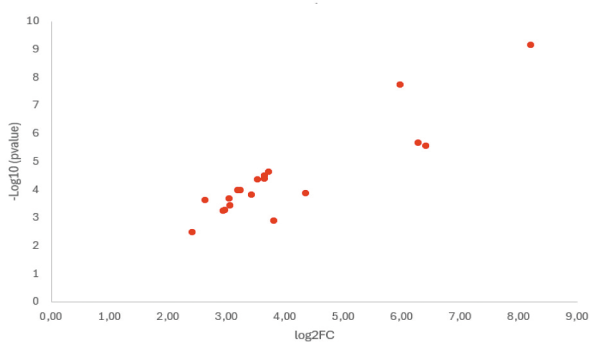

Genename	log2FoldChange
PCDHB2	8,21
PCDHB5	6,42
PCDHB3	6,28
PCDHB1-AS1	5,97
PCDHB10	4,35
PCDHB4	3,82
PCDHA1	3,73
PCDHA2	3,65
PCDHA3	3,65

PCDHA4	3,53
PCDHB9	3,44
PCDHA5	3,24
PCDHA6	3,19
PCDHA9	3,06
PCDHA7	3,05
PCDHA10	2,97
PCDHA8	2,94
PCDHB13	2,64
PCDHA11	2,42
