# Supplementary material for: Splenomegaly in de novo acute myeloid leukemia is associated with ASXL1 mutations together with a distinct clinical and gene expression profile
Source: Biomark Res. 2025 Oct 22;13:131. doi: 10.1186/s40364-025-00833-8 (PMC12542016; doi:10.1186/s40364-025-00833-8)
Supplement: Supplementary file 2 — Supplementary Material 2: Figure 1. [file 40364_2025_833_MOESM2_ESM.docx]

**Supplementary File 1**

**Materials and methods**

**Patients**

From 2005 to 2022, 560 patients aged ≥ 18 years were diagnosed with *de novo* acute myeloid leukemia (AML) at our institution. Informed consent was obtained from all patients before study inclusion, in accordance with the Declaration of Helsinki. The local Ethics Committee approved the study. Patients records/information were anonymized and de-identified before analysis.

**Spleen size evaluation**

All patients underwent abdominal ultrasound to evaluate spleen size during the routine AML diagnostic work-up procedures. According to the available evidence, splenomegaly was defined as a splenic diameter larger than 12 cm at ultrasound (US) evaluation [1–3]. For AML patients included in this study, other possible causes of splenomegaly (i.e., active or chronic infections, portal hypertension, solid cancer) were consequently ruled out. None of the AML patients had a prior history or clinical evidence of a pre-existing myeloproliferative neoplasm.

**Morphological and immunophenotypic assessment**

Two independent hematologists evaluated both PB and BM smears of all patients to make the morphological definition of AML according to the FAB classification [4]. A comprehensive flow cytometric workup was performed on BM and PB samples. The total markers assessed included CD2, surface CD3, cytoplasmic CD3, CD4, CD5, CD7, CD8, CD9, CD10, CD11b, CD11c, CD13, CD14, CD15, CD16, CD19, CD20, CD22, CD33, CD34, CD38, CD41a, CD56, CD45, CD64, cytoplasmic CD79a, CD117, CD123, CD235, NG2, MPO, HLA‐DR, terminal deoxynucleotidyl transferase (TDT) [5].

***ASXL1* mutational analysis**

The genomic DNA was extracted from the BM samples at onset using the QIAamp DNA Blood Mini Kit (Qiagen, Valencia, CA, USA). DNA quantification was performed using a QUBIT®2.0 Fluorometer (Invitrogen). Somatic mutations of *ASXL1* genes were identified by sequencing after polymerase chain reaction (PCR) amplification of genomic DNA [6]. PCR amplifications were done in a total volume of 25 ul PCR mix containing at least 250ng template DNA, Taq buffer, 200 umol of each deoxynucleotide triphosphate, 20 pmol of each primer and 1 unit of Platinum Taq DNA Polymerase (Life Technologies). PCR amplification conditions were as follows: 95°C 10 min; 95°C 30 s, 55°C 30 s, 72°C 30 s to 1 min for 35 cycles; 72°C 10 min. PCR products were purified using the QIAquick Gel Extraction Kit (Qiagen, Valencia, CA, USA). Purified PCR products were used for sequencing using the Big Dye terminator v1.1 kit (Life Technologies) including the forward or reverse primer. After purification with BigDye Xterminator Purification Kit, sequences were loaded on an SeqStudio Genetic Analyzer (Life Technologies). The sequence data files were analyzed using Sequencing Analysis Software v6.0 (Life Technologies).

**Targeted next-generation sequencing (NGS)**

With the aim of gaining a more in-depth knowledge of the molecular profile of AML cases with an enlarged spleen volume (45 of the 58 cases yielded sufficient material), NGS analysis was performed on the same BM genomic DNA extracted for direct sequencing, using an AmpliSeq customized panel (Thermo Fisher) encompassing the full coding regions or specific exons of 26 target genes involved in the pathogenesis of myeloid malignancies (*ANKRD26, ASXL1, CALR, CBL, CEBPA, DDX41, DNMT3A, ETV6, EZH2, FLT3, GATA2, IDH1, IDH2, JAK2, KIT, KRAS, MPL, NPM1, NRAS, RUNX1, SF3B1, SRSF2, TET2, TP53, U2AF1, ZRSR2*), as previously reported [7–9]. Torrent Suite Software (Thermo Fisher) was used for quality control, alignment to the human genome (hg19) and variant calling, using the somatic workflow for single samples with the default parameters. Variants were annotated with Ion Reporter Software (Thermo Fisher). Variants located in intronic regions, synonymous or those present with >1% global minor allele frequency (MAF) in the normal population were filtered out. The selected variants were investigated for a potential pathogenic role using the SIFT and PolyPhen scores and the Catalogue Of Somatic Mutations In Cancer (COSMIC) and ClinVAR databases.

**Gene expression profiling**

To compare the transcriptomics profiles of AML patients with and without *ASXL1* mutations, gene expression profiling was performed by RNA-sequencing (RNAseq) on the Illumina NovaSeq6000 platform. Total RNA was extracted from 13 AML patients with splenomegaly carrying *ASXL1*mut and 7 AML patients, with (4/7) or without (3/7) splenomegaly, with no identified *ASXL1* mutations (*ASXL1*wt). Transcriptomic libraries were prepared using the Illumina TruSeq Stranded Total RNA kit. Samples processing was automatized using the MicroLab STAR® automated system (Hamilton Company). The prepared libraries were sequenced on the Illumina NovaSeq6000 platform.

Paired-end reads fastq files were quality checked using FastQC version 0.12.1. Multiple sample quality control results were summarized with MultiQC [10]. Sequencing adapters trimming was performed by Trimmomatic tool version 0.32 [11]. Post-trimming quality control check was performed by FastQC and MultiQC. Trimmed reads were mapped against the Homo sapiens reference genome (GRCh.38: https://ftp.ensembl.org/pub/release-109/fasta/homo_sapiens/dna/ ) using the STAR aligner [12]. The FeatureCounts [13] algorithm was used to successfully assign mapped reads on a single gene following tgenome annotation (version 109) from the Ensembl database(https://ftp.ensembl.org/pub/release109/gtf/homo_sapiens/Homo_sapiens.GRCh38.109.gtf.gz). Principal Component Analysis (PCA) was performed on genes expressed in the DESeq2 R package [14] to illustrate the variability and investigate similarities between subjects with and without *ASXL1* mutations. Detection and comparison of differentially expressed genes between groups was performed by DESeq2. Data are visualized on the volcano plot. Gene names and gene ontology annotations (MP: Molecular Function, CC: Cellular Component, BP: Biological Process) of differentially expressed genes (DEGs) were retrieved from Human genome annotation resource (available from org.Hs.eg.db R package [15]) in combination with AnnotationHub and AnnotationDbi R packages [16,17]. Data were integrated using the Database for Annotation, Visualization and Integrated Discovery (DAVID) v2024q2 (July 2024) [18,19].

DEGs were used to query Ingenuity Pathways Analysis (IPA) software (QIAGEN, Hilden, Germany) to identify significantly regulated functional pathways. The ‘core analysis’ function of IPA was performed to identify canonical pathways, networks and upstream regulators [20].

**Statistical analysis**

Statistical analyses were carried out using GraphPad Prism version 8.3.0 (GraphPad Software Inc., San Diego, CA, USA). Continuous variables are presented as median, minimum–maximum, and interquartile range (IQR). The Shapiro-Wilk test was performed to check the normal distribution of continuous variables. The differences in the distribution of continuous variables between categories were compared using *t* test, Mann-Whitney, ANOVA or Kruskal-Wallis test. Survival analysis was performed using the Kaplan–Meier method, and differences between groups were compared using the log-rank test. A *p* value < 0.05 was considered statistically significant.

Mutual exclusivity analysis between gene variants detected from the NGS analysis was performed using the online cBioPortal Oncoprinter tool (https://www.cbioportal.org/oncoprinter).

**Results**

**Gene expression profiling**

To verify whether the co-occurrence of an enlarged spleen volume and *ASXL1* mutations in AML is linked to a specific transcriptional profile, high-throughput RNA sequencing was conducted. Gene expression profiles from 13/23 *ASXL1*mut cases (with splenomegaly) were compared with 7 *ASXL1*wt patients (4 with and 3 without splenomegaly). According to the PCA results (Figure 2G), carried out on read counts relative to the expressed genes, a clear separation was observed between the two groups. The *ASXL1*wt group showed a substantial homogeneity between samples regardless of the splenomegaly condition (Supplementary Figure 2); therefore, gene expression profiles of this group were considered as control in the subsequent analysis.

Comparing the gene expression profiles of the *ASXL1*wt and *ASXL1*mut groups, a total of 1495 DEGs were detected, 811 protein coding genes (642 up-regulated and 169 down-regulated) and 684 elements (481 up and 203 down) belonging to other categories (lncRNAs, pseudogenes, etc.). The complete list of DEGs is reported in Supplementary Table 2. A prioritized list of DEGs was created considering a log-fold-change (Log2FC) of more than 1.5 or less than -1.5 with a p-adjusted value lower than 0.05. This revealed 578 differentially expressed protein coding genes (514 up and 64 down) and 501 elements belonging to other categories (416 up and 85 down) between the two groups (Supplementary Table 3). Selected DEGs have been visualized on volcano plots (Supplementary Figure 3A).

To understand the effects of different gene expressions between *ASXL1*mut and *ASXL1*wt patients, statistically significant DE protein coding genes were categorized following KEGG pathways and Gene Ontology (GO) annotations. KEGG pathways analysis evidenced that the networks linked to neuromuscular transmission and muscle architecture were the most significantly represented (Supplementary Table 4).

Interestingly, pathways related to signal (synaptic) transmission and nervous system development also resulted the most significantly represented, in the TOP Biological Process (BP) category. At the same time, categories related to cell-cell interactions and plasma membrane structures resulted the most significantly represented in the TOP Cellular Components (CC) category. Finally, cellular elements related to extracellular molecules resulted the most significantly represented in the TOP Molecular Functions (MF) category (Supplementary Figure 3B). The complete list of gene ontology categories (BP, MF, CC) with the categorized genes is reported in Supplementary Table 5.

To highlight the most significantly altered molecular mechanisms between groups, DEGs were used to query IPA software (QIAGEN™). A strong enrichment in canonical pathways was observed in “Neurexins and neuroligins” (p=4.73E-06) with 8 DEGs, in “Regulation of Insulin-like growth factor (IGF) transport and uptake by IGFBPs” (p=8.08E-06) with 11 DEGs, and in “Cohesin Chromatin Regulation Pathway” (p=1.13E-05) with 16 DEGs (Supplementary Figure 3C and Supplementary Table 6). The complete list of canonical pathways evidenced from the analysis is reported in Supplementary Table 7.

Analysing the biofunctions in which the DEGs are involved, significant correlations were emphasized with cardiotoxicity, hepatotoxicity, and nephrotoxicity. In agreement with GO analysis, the involvement of molecules linked to cellular development, growth/proliferation, morphology, assembly/organization and function/maintenance as well as involved in nervous system development/function, organismal development, tissue development and organismal survival was observed.

At a deeper transcript level regulation, *PCDHB2* gene resulted the TOP UPregulated DEG (Log2FC=8.211 and p-adjusted value 4.74e-07) (Figure 2H). The protein is a member of the protocadherin (PCDH) family and is a calcium-dependent cell-adhesion protein linked to the establishment and maintenance of specific neuronal connections in the brain.

It is very interesting to focus attention on data regarding the PCDH family, that play crucial roles in cell signalling and development [21]. Indeed, beyond *PCDHB2*, many other members of the protocadherin family, at least 18 coding genes and 1 lncRNA (*PCDHB1-AS1*), resulted significantly upregulated in the *ASXL1*mut group (Supplementary Figure 3D). These data would suggest a possible correlation with the dysregulation of cell signalling.

In our analysis, both the couple leucine-rich adaptor protein 1-like (*LURAP1L)* and *LURAP1L* antisense RNA 1 *(LURAP1L-AS1)* resulted upregulated (7.71 and 7.97 times) in *ASXL1*mut patients, highlighting another important aspect in this study. The *LURAP1L* gene encodes for a protein involved in the regulation of signal transduction [22], suggesting that deregulated pathway mechanisms may possibly be correlated with AML onset in *ASXL1* mutated samples.

Paying attention to the most significantly downregulated transcripts, *POU4F1* is the TOP DOWNregulated DEG (Log2FC=-6.231 and p-adjusted value = 8.78e-07) (Figure 2H). It is a transcription factor that regulates the expression of specific genes involved in differentiation and survival within a subset of neuronal lineages.

All these data indicate a direct action of *ASXL1* mutations on key networks for signal transmission and cellular communication linked to neuronal development and function that need to be further explored and investigated.

**References**

1. Arora N, Sharma PK, Sahai A, Singh R. Sonographic measurement of the spleen: Splenic length in adults and its correlation with different parameters. J Anat Soc India [Internet]. Anatomical Society of India; 2013;62:57–61. Available from: http://dx.doi.org/10.1016/S0003-2778(13)80014-7

2. Lamb PM, Lund A, Kanagasabay RR, Martin A, Webb JAW, Reznek RH. Spleen size: How well do linear ultrasound measurements correlate with three-dimensional CT volume assessments? Br J Radiol. 2002;75:573–7.

3. Niederau C, Sonnenberg A, Muller JE, Erckenbrecht JF, Scholten T, Fritsch WP. Sonographic measurements of the normal liver, spleen, pancreas, and portal vein. Radiology. 1983;149:537–40.

4. Bennett JM, Catovsky D, Daniel M ‐T, Flandrin G, Galton DAG, Gralnick HR, et al. Proposals for the Classification of the Acute Leukaemias French‐American‐British (FAB) Co‐operative Group. Br J Haematol. 1976;33:451–8.

5. Wood BL, Arroz M, Barnett D, DiGiuseppe J, Greig B, Kussick SJ, et al. 2006 Bethesda International Consensus recommendations on the immunophenotypic analysis of hematolymphoid neoplasia by flow cytometry: Optimal reagents and reporting for the flow cytometric diagnosis of hematopoietic neoplasia. Cytom Part B - Clin Cytom. 2007;72:14–22.

6. Gelsi-Boyer V, Trouplin V, Adélaïde J, Bonansea J, Cervera N, Carbuccia N, et al. Mutations of polycomb-associated gene ASXL1 in myelodysplastic syndromes and chronic myelomonocytic leukaemia. Br J Haematol. 2009;145:788–800.

7. Cumbo C, Tarantini F, Anelli L, Zagaria A, Redavid I, Minervini CF, et al. IRF4 expression is low in Philadelphia negative myeloproliferative neoplasms and is associated with a worse prognosis. Exp Hematol Oncol [Internet]. BioMed Central; 2021;10:1–4. Available from: https://doi.org/10.1186/s40164-021-00253-y

8. Cumbo C, Tarantini F, Zagaria A, Anelli L, Minervini CF, Coccaro N, et al. Clonal Hematopoiesis at the Crossroads of Inflammatory Bowel Diseases and Hematological Malignancies: A Biological Link? Front Oncol. 2022;12:1–5.

9. Cumbo C, Orsini P, Tarantini F, Anelli L, Zagaria A, Tragni V, et al. TNFRSF13B gene mutation in familial acute myeloid leukemia: A new piece in the complex scenario of hereditary predisposition? Hematol Oncol. 2023;41:942–6.

10. Ewels P, Magnusson M, Lundin S, Käller M. MultiQC: Summarize analysis results for multiple tools and samples in a single report. Bioinformatics. 2016;32:3047–8.

11. Bolger AM, Lohse M, Usadel B. Trimmomatic: A flexible trimmer for Illumina sequence data. Bioinformatics. 2014;30:2114–20.

12. Dobin A, Davis CA, Schlesinger F, Drenkow J, Zaleski C, Jha S, et al. STAR: Ultrafast universal RNA-seq aligner. Bioinformatics. 2013;29:15–21.

13. Liao Y, Smyth GK, Shi W. FeatureCounts: An efficient general purpose program for assigning sequence reads to genomic features. Bioinformatics. 2014;30:923–30.

14. Michael A, Forbes K. Package ‘ DESeq2 .’ 2025;

15. Also S. org.Hs.eg.db. 2025;

16. Carlson M, Morrell K. Package ‘ AnnotationHub .’ 2025;

17. R FRARBRG. Package ‘ AnnotationDbi .’ 2025;

18. Sherman BT, Hao M, Qiu J, Jiao X, Baseler MW, Lane HC, et al. DAVID: a web server for functional enrichment analysis and functional annotation of gene lists (2021 update). Nucleic Acids Res. Oxford University Press; 2022;50:W216–21.

19. Huang DW, Sherman BT, Lempicki RA. Systematic and integrative analysis of large gene lists using DAVID bioinformatics resources. Nat Protoc. 2009;4:44–57.

20. Krämer A, Green J, Pollard J, Tugendreich S. Causal analysis approaches in ingenuity pathway analysis. Bioinformatics. 2014;30:523–30.

21. Chen W V., Maniatis T. Clustered protocadherins. Dev. 2013;140:3297–302.

22. Lee ICJ, Leung T, Tan I. Adaptor protein LRAP25 mediates myotonic dystrophy kinase-related Cdc42-binding kinase (MRCK) regulation of LIMK1 protein in lamellipodial F-actin dynamics. J Biol Chem. 2014;289:26989–7003.
