# Supplementary material for: Splenomegaly in de novo acute myeloid leukemia is associated with ASXL1 mutations together with a distinct clinical and gene expression profile
Source: Biomark Res. 2025 Oct 22;13:131. doi: 10.1186/s40364-025-00833-8 (PMC12542016; doi:10.1186/s40364-025-00833-8)
Supplement: Supplementary file 3 — Supplementary Material 3: Figure 2. [file 40364_2025_833_MOESM3_ESM.docx]

**Supplementary Table 1.** AML patients main biological and clinical characteristics.

|  | **AML (n=58)** |
| --- | --- |
| **Gender M/F (%)** | 44/14 (76/24) |
| **Median age, years (range)** | 65 (25-88) |
| **Median WBC/uL (range)** | 27800 (870-460600) |
| **Median Hb g/dL (range)** | 8.7 (5.3-14.5) |
| **Median PLT/uL (range)** | 106000 (4220-947000) |
| **Median BM blasts %, (range)** | 56 (23-95) |
| **Median spleen diameter, cm (range), by US** | 14 (13-22) |
| **FAB subtype, N (%)** | M0, 4 (7); M1, 2 (3); M2, 21 (36); M4, 24 (42); M5, 7 (12) |
| **ELN risk, low/intermediate/high (%)** | 2/27/29 (3/47/50) |
| **Lines of therapy, 1/+1 (%)** | 42/16 (72/28) |
| **Type of therapy, ICT/LIT/BSC (%)** | 43/7/8 (74/12/14) |
| **HSCT, Y/N (%)** | 7/51 (12/88) |
| **Median OS, months (range)** | 9.5 (1-109) |

AML: acute myeloid leukemia, WBC: white blood cells, Hb: hemoglobin, PLT: platelets, BM: bone marrow, US: ultrasound, FAB: French-American-British Classification, ELN: European Leukemia Net, ICT: intensive chemotherapy, LIT: low-intensive therapy, BSC: best supportive care, HSCT: hematopoietic stem cells transplant, OS: overall survival.
