# Supplementary figures and images for: Splenomegaly in de novo acute myeloid leukemia is associated with ASXL1 mutations together with a distinct clinical and gene expression profile
Source: Biomark Res. 2025 Oct 22;13:131. doi: 10.1186/s40364-025-00833-8 (PMC12542016; doi:10.1186/s40364-025-00833-8)

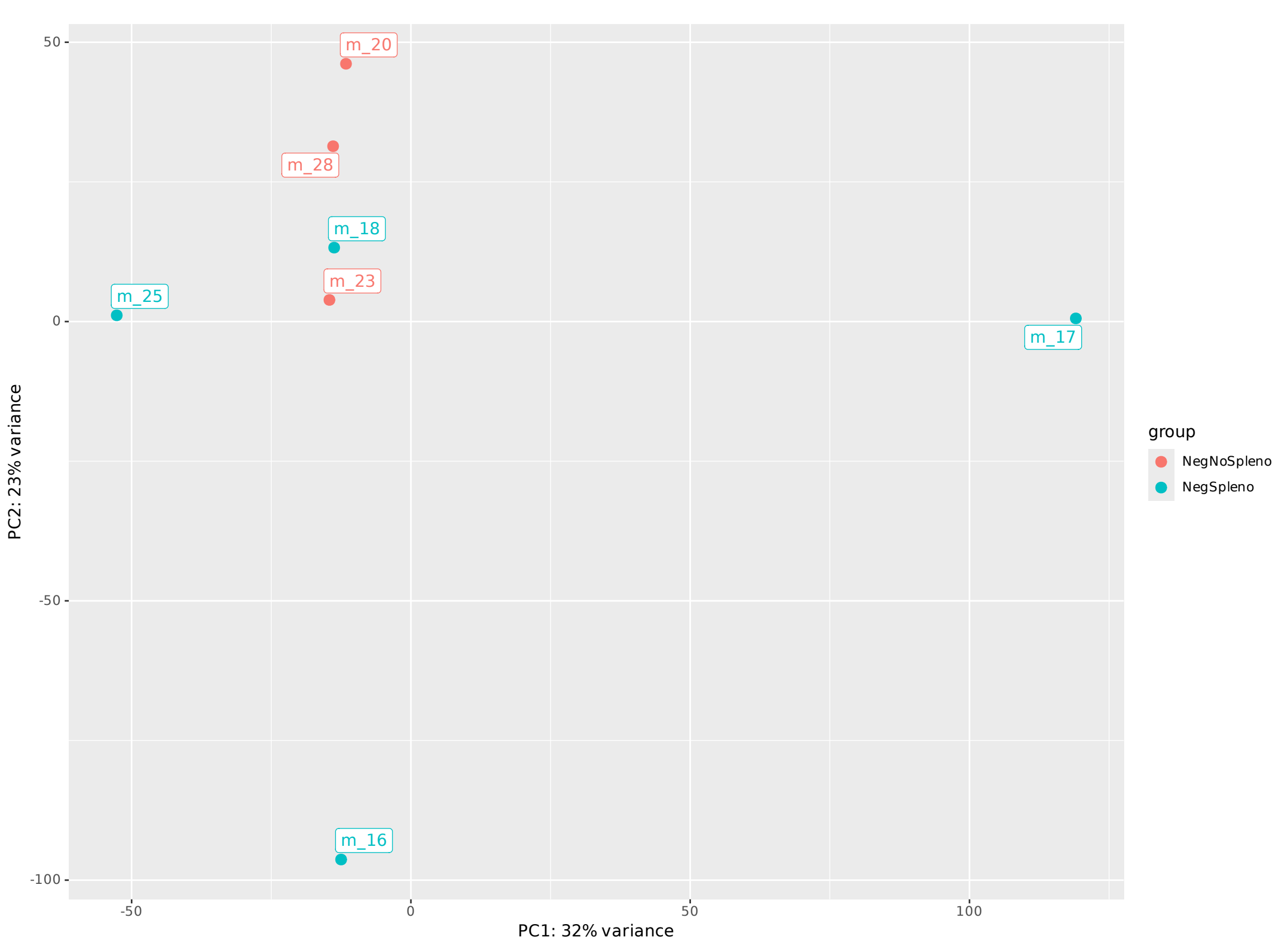

Supplement: Supplementary file 10 — Supplementary Material 10: Table 7. [file 40364_2025_833_MOESM10_ESM.pdf]
